# Lysine demethylase 2A expression in cancer-associated fibroblasts promotes breast tumour growth

**DOI:** 10.1038/s41416-020-01112-z

**Published:** 2020-10-07

**Authors:** Jing-Yi Chen, Chien-Feng Li, You-Syuan Lai, Wen-Chun Hung

**Affiliations:** 1 School of Medicine for International Students, College of Medicine, I-Shou University, 840 Kaohsiung, Taiwan; 2 Department of Pathology, Chi-Mei Foundation Medical Center, 710 Tainan, Taiwan; 3 National Institute of Cancer Research, National Health Research Institutes, 704 Tainan, Taiwan; 4 Graduate Institute of Medicine, College of Medicine, Kaohsiung Medical University, 807 Kaohsiung, Taiwan; 5 Drug Development and Value Creation Research Center, Kaohsiung Medical University, 807 Kaohsiung, Taiwan; 6 Department of Medical Research, Kaohsiung Medical University Hospital, 807 Kaohsiung, Taiwan

**Keywords:** Breast cancer, Senescence

## Abstract

**Background:**

Our previous study demonstrated that lysine demethylase 2A (KDM2A) enhances stemness in breast cancer cells. This demethylase is also highly expressed in cancer-associated fibroblasts (CAFs). However, its clinical significance is unclear.

**Methods:**

The expression of KDM2A in CAFs was studied using immunohistochemical staining and its association with clinicopathological features and patient’s survival was tested. Overexpression and knockdown strategies were used to investigate KDM2A-regulated genes in fibroblasts. Senescent cells were detected by using β-galactosidase staining. The *in vivo* tumour-promoting activity of stromal KDM2A was confirmed by animal study.

**Results:**

Increase of stromal KDM2A is associated with advanced tumour stage and poor clinical outcome in breast cancer patients. Cancer-derived cytokines stimulated KDM2A expression in normal fibroblasts and transformed them into CAFs. Upregulation of KDM2A induced p53-dependent senescence in fibroblasts and enhanced the release of cytokines, which reciprocally promoted cancer cell proliferation. Additionally, KDM2A upregulated programmed death-ligand 1 (PD-L1) expression via transcriptional activation in fibroblasts. Knockdown of KDM2A completely abolished the tumour-promoting activity of CAFs on breast tumour growth in vivo and diminished PD-L1 expression in the stroma of tumour tissues.

**Conclusions:**

Stromal KDM2A plays an oncogenic role in breast cancer and inhibition of KDM2A reduces fibroblast senescence and suppresses tumour growth.

## Background

Post-translational modifications (PTMs) of histone proteins modulate chromatin structure, DNA-histone interaction and transcription factor recruitment that affect gene expression under various physiological and pathological circumstances.^1,2^ Lysine methylation is one of the most prevalent PTMs occurred in histone proteins and is dynamically controlled by lysine methyltransferases (KMTs) and demethylases (KDMs). More than 30 members of KMTs have been identified in mammalian cells.^3–5^ Several lysine residues like Lys4 histone H3 (H3K4) are known to be methylated by multiple KMTs and the enzymes responsive for the introduction of mono-, di- and trimethylation are different. Conversely, H3K79 can only be methylated by KMT4 (also known as DOT1L) with high specificity. Similar to KMTs, more than 20 KDMs are existed in human genome. The erasure of lysine methylation on different histone proteins like H3K4, H3K9 and H3K27 is also achieved by multiple KDMs. However, the KDMs responsive for the demethylation of H3K79 and H4K20 are still unclear.

Lysine demethylase 2A (KDM2A) was originally identified as an F-box motif-containing mammalian protein, and is a component of the SCF ubiquitin ligase.^6^ By using biochemical purification, Tsukada et al. isolated a H3K36 demethylase with the unique JmjC domain and named it as JmjC domain-containing histone demethylase 1 (JHDM1).^7^ Subsequent studies confirmed that these two molecules are encoded by the same gene, which is now officially nominated as KDM2A. The first biological function of KDM2A reported was the regulation of cell proliferation and senescence by modulating p53 and p15 expression.^8,9^ Upon starvation, KDM2A was found to bind to ribosomal DNA promoter to suppress gene expression via a demethylase activity-dependent mechanism.^10^ In addition to demethylate H3K36, KDM2A can erase the methyl group from non-histone proteins like NF-kB to modulate intracellular signalling and inflammatory response.^11,12^

The involvement of KDM2A in tumorigenesis was firstly demonstrated in lung cancer. Wagner et al. showed that KDM2A is overexpressed in lung tumours, and its upregulation induces promoter demethylation of the dual-specificity phosphatase 3 gene, which leads to constitutive activation of extracellular signal-regulated kinase 1 (ERK1) and ERK2 signalling to promote tumour formation.^13^ Our previous study showed that KDM2A is increased in breast cancer and is associated with poor patient’s survival.^14^ Knockdown of KDM2A in breast cancer cells reduced the expression of NOTCH signalling molecules including Jagged1, NOTCH1 and HEY1, and attenuated cancer stemness and angiogenesis. These results suggested that KDM2A plays an oncogenic role in breast cancer. Recently, we found that expression of KDM2A is inversely correlated with the level of the tumour suppressor tet-eleven translocation 2 (TET2), a DNA demethylase, in breast cancer.^15^ Our data demonstrated that KDM2A suppresses TET2 expression via transcriptional repression, which leads to the reduction of two TET2 target genes, E-cadherin and epithelial cell adhesion molecule, and the increase of cancer invasiveness. Our study provides a novel mechanism by which a histone demethylase (KDM2A) may control DNA methylation by regulating the expression of a DNA demethylase (TET2). Interestingly, when we checked KDM2A expression in human breast tumour tissues, we found that KDM2A is also highly upregulated in CAFs. In this study, we investigated the clinical significance of KDM2A in tumour stroma and tried to elucidate the underlying mechanism by which stromal KDM2A promotes breast tumorigenesis.

## Methods

### Patients and tissue sections

Study of human tumour tissues was approved by the Institutional Review Board Committee of Chi-Mei Foundation Medical Center (No. 10210004). Written informed consents were obtained from all patients participated. The tissues slides were stained with anti-KDM2A antibody and the staining intensity levels were calculated using the H-score, defined by the following equation: H-score = ΣPi (i + 1) as previously described.^16^ I is the intensity of the stained tumour cells (0 to 3+), and Pi is the percentage of positive-stained tumour cells. Kaplan–Meier plots was used in overall survival analyses.

### Cell culture and reagents

Immortalised normal human breast fibroblasts RMF-EG cell line was originally established by Dr. Charlotte Kuperwasser (Tufts University, Boston, MA, USA)^17^ and was kindly supplied from Dr. Kelvin Kun-Chih Tsai (Graduate Institute of Clinical Medicine, Taipei Medical University, Taipei) with permission. MDA-MB-231 and MCF-7 cell lines were purchased from the Bioresource Collection and Research Center (BCRC) and cell identity was certified by short tandem repeat analysis. The RMF-EG, MDA-MB-231 and MCF-7 cells were cultured in Dulbecco’s Modified Eagle’s medium (DMEM) containing 10% foetal bovine serum (FBS). The KDM2A inhibitor daminozide was purchased from Cayman (Ann Arbor, MI, USA). Anti-KDM2A (clone ab240727) and anti-PD-L1 antibody conjugated with Alexa Fluor 488 (clone ab209959) antibodies were obtained from Abcam (Cambridge, MA, USA). Anti-p53 (clone DO-1) and anti-actin (clone H-2) antibodies were purchased from Santa Cruz Biotechnology, Inc. (Dallas, TX, USA). Anti-phospho-histone-H2A.X (Ser139) (clone JBW301) was purchased from Millipore Corporation (Billerica, MA, USA). Anti-p38 mitogen-activated protein kinase (MAPK), anti-phospho-p38 MAPK (Thr180/Tyr182), anti-phospho-Chk1 (Ser317), anti-phospho-Chk2 (Thr68) (clone C13C1), anti-histone H3K36me2 (clone C75H12), anti-histone H3K36me3 (clone D5A7) and anti-histone H3K4me3 (clone 9727) antibodies were obtained from Cell Signaling Technology Inc. (Danvers, MA, USA). Anti-PD-L1 (clone GTX01495), anti-FAP (clone GTX108711) and anti-PDGFR-α (clone GTX01102) antibodies were obtained from GeneTex Inc. (Hsinchu, Taiwan).

### Promoter activity assay

RMF-EG cells were seeded in 24-well plates and transfected with pEZX-PG04-KDM2A promoter construct-linked Gaussia luciferase (GLuc) and secreted alkaline phosphatase (SEAP) reporter plasmid (GeneCopoeia, Inc., Rockville, MD, USA). After transfection, the cells were treated with recombinant human interlukin-6 (IL-6) and tumour necrotic factor-α (TNF-α) (20 ng/ml) for 24 h. Measurement of luciferase activities was determined using the Secrete-Pair Dual Luminescence Assay Kit (GeneCopoeia, Inc., Rockville, MD, USA). The Gaussia luciferase activity was measured by using a Luminometer and was normalised with SEAP and total protein concentration.

### Establishment of stable cell lines

The pLKO.shRNA-KDM2A was purchased from the National Core Facility for Manipulation of Gene Function by RNAi, miRNA, miRNA sponges and CRISPR/Genomic Research Center (Academia Sinica, Taipei). KDM2A expression vector was kindly supplied from Dr. Yi Zhang (Harvard University, MA, USA). The plasmid was transfected into RMF-EG cells using the Neon^TM^ microporation transfection system. RMF-EG cells (1 × 10^6^) were resuspended in buffer R containing 1 μg plasmid and subjected to electroporation at 1800 V, 3 mini-second and 2 pulses. After electroporation, the cells were cultured at 37 °C in an incubator with 5% CO_2_-humidified atmosphere. The KDM2A-silenced stable cell line (RMF-shKDM2A) was selected by 1 μg/ml puromycin. KDM2A-expressing stable cell lines K2A-1 and K2A-2 were selected by 800 μg/ml G418. The KDM2A protein levels in stable cell lines were detected by western blotting.

### Co-culture of fibroblasts with breast cancer cells

RMF-EG cells were co-cultured with breast cancer cells using transwell system (Corning, NY, USA). RMF-EG cells (1 × 10^5^) were grown in the lower chamber, and breast cancer cells (1 × 10^5^) were seeded on the polyester membrane (0.4 µm) of a transwell insert in DMEM medium containing 10% FBS. The cells were incubated in a humidified 5% CO_2_ incubator at 37 °C for 72 h and were harvested for different assays.

### Western blotting

Total proteins were extracted from cells with RIPA buffer (50 mM Tris-HCl, pH 7.4, 150 mM NaCl, 1% NP-40, 0.1% SDS, 0.5% sodium deoxycholate, 2 mM EDTA and 50 mM NaF) containing protease inhibitors. The concentrations of cellular proteins were determined by Bradford assay. Forty microgram of cellular proteins were separated by SDS-polyacrylamide gel electrophoresis. Proteins were transferred to polyvinylidene difluoride (PVDF) membranes and probed with various primary and secondary antibodies. Finally, the signals on the membranes were developed by enhanced chemiluminescence reagent.

### RNA extraction and quantitative reverse transcription-polymerase chain reaction (qRT-PCR) analysis

Cells (1 × 10^6^) were harvested and RNA was extracted using an RNA extraction kit (Geneaid, New Taipei City, Taiwan). One microgram of RNA was converted into cDNA by Moloney murine leukaemia virus (M-MLV) reverse transcriptase (Promega Corporation, Fitchburg, WI, USA) and the expression of target mRNAs was quantified using real-time PCR reactions with SYBR green fluorescein as previously described.^15^ Primer sequences were shown in Table S1.

#### Chromatin immunoprecipitation assay-quantitative PCR (ChIP-qPCR)

Cells were cross-linked in 3.7% formaldehyde solution for 10 min at 37 °C and incubated in a lysis buffer (1% SDS, 10 mM EDTA, 50 mM Tris-HCl, pH 8.1) for 10 min on ice. Genomic DNA was sonicated with pulse of 15 sec at 3 µm amplitude to obtain an average fragment size between 500 and 1000 bp. DNA-protein complexes were immunoprecipitated by antibodies against the rabbit IgG, KDM2A, histone H3K36me2, H3K36me3 or H3K4me3. DNA fragments were recovered and quantified by quantitative PCR using specific primers for the detection of the CpG islands upstream of PD-L1 transcription start site predicted by using the University of California Santa Cruz genome browser (https://genome.ucsc.edu). Primer sequences used for chromatin immunoprecipitation assay was showed in Supplementary table S1. ChIP-qPCR was performed to confirm relative enrichment of specific sequences within specific antibodies precipitated DNA relative to the DNA fragment precipitated by rabbit IgG as negative controls to normalise enrichment of each positive amplicon.

### Senescence assay

Senescent cells were detected by using Senescence β-Galactosidase staining kit (Cell Signaling Technology Inc.). The cells were washed with PBS, fixed with fixation solution for 10 min at room temperature and incubated with β-galactosidase staining solution at 37 °C overnight. After incubation, cells with blue colour were counted in five randomly selected fields under a microscope and the percentage of positive-stained cells was shown as Mean ± SE from three independent experiments.

### Orthotopic animal study

RMF-EG or RMF-shKDM2A cells (5 × 10^6^) were mixed with MDA-MB-231 cells (5 × 10^6^) in 100 μl Hank’s balanced salt solution containing 0.5% Matrigel (BD Biosciences, San Jose, CA, USA). The cells were inoculated into the fourth mammary fat pads of 6-week-old female BALB/cAnN.Cg-Foxn1nu/CrlNarl mice (*n* = 5 for each experimental group). One week after cell inoculation, tumour growth was monitored and tumour volume was calculated using the equation: tumour volume = (length × width^2^)/2. After 3 weeks, tumour-bearing animals were euthanised by isoflurane inhalation and euthanised by cervical dislocation. The tumours were isolated and tumour weights were measured. The statistical difference between experimental groups was evaluated by *t*-test. Animal use protocol was approved (No. IACUC-106110) by the Institutional Animal Care and Use Committee of National Health Research Institutes.

### Immunohistochemical staining

The expression of KDM2A and PD-L1 proteins was detected by using the immunostaining procedure previously described.^14^ The quantification of PD-L1- or GLB1-positive CAFs were determined by the mean of four independent views under the microscope. Data are expressed as Mean ± SD from five animals per experimental group.

#### Flow cytometry

RMF-EG and RMF-KDM2A cells were fixed with 3.7% formaldehyde for 15 min at room temperature. After washing, cells were incubated with anti-PD-L1 antibody conjugated with Alexa Fluor 488 for 1 h at room temperature. Cells were washed and the PD-L1-positive cells were detected with Attune NxT Flow Cytometer (Invitrogen).

### γ-H2AX detection

Cells were fixed for 20 min at room temperature with 2% paraformaldehyde. After washing, cells were permeabilised with pre-chilled ethanol 70%, and stored at 4 °C overnight. Cells were washed with PBS and blocked with 5% BSA in PBS containing 0.5% Tween-20 and 0.1% Triton X-100 for 30 min at room temperature. After blocking, cells were incubated at 4 °C overnight with anti-phosph-histone-H2A.X (Ser139) antibody, followed by incubation of the donkey anti-mouse Alexa-488-conjugated IgG. After final washing, the samples were counter-stained with 4’,6-diamidino-2-phenylindole (DAPI). The slides were mounted using mounting medium for fluorescence and observed under the Leica CTR4000 microscope.

### Statistical analysis

All experiments were done in triplicate and were repeated two to three times. The *t*-test was used to compare independent experimental groups in in vitro and in vivo experiments. Two-tailed *P* values ≤0.05 were considered statistically significant. Statistical analysis was performed using the GraphPad Prism version 5.01 (GraphPad Software, Inc.).

## Results

### Upregulation of KDM2A in CAFs is associated with advanced tumour stage and poor patient’s outcome

**Fig. 1:**
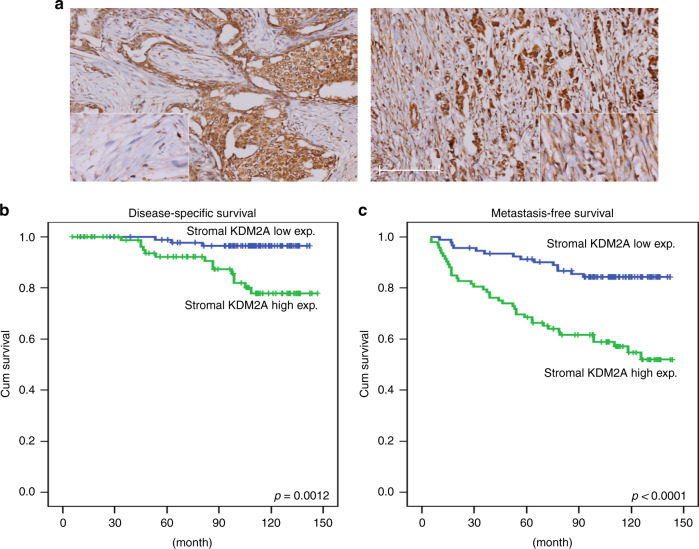
KDM2A expression in tumour stroma is associated with poor survival of breast cancer patients. **a** Expression of KDM2A in breast tumour stroma was detected by immunohistochemical staining and the tumours with low (left) and high (right) expression were shown. **b** Disease-specific survival of 184 breast cancer patients with low or high KDM2A was compared. *P* = 0.0012. **c** Metastasis-free survival of 184 breast cancer patients with low or high KDM2A was compared. *P* < 0.0001.

Immunohistochemical staining showed that KDM2A was highly expressed in some breast tumour tissues (Fig. 1a). In addition, KDM2A protein level was increased in the CAFs isolated from tumour tissue comparing with the normal fibroblasts isolated from non-tumour part (Fig. S1). Clinicopathological association analysis demonstrated that stromal KDM2A expression was correlated with increased tumour size, lymph node invasion, clinical stage and histological grade (Table 1). In addition, breast cancer patients with strong stromal KDM2A expression have a shorter disease-specific survival (DSS) (*p* = 0.0012) and metastasis-free survival (MeFS) (*p* < 0.0001) (Fig. 1b, c). Univariate log-rank analysis for DSS and MeFS showed that high stromal KDM2A was associated with increased tumour size, lymph node invasion and clinical stage (Table S2). Multivariate survival analysis also demonstrated a worse survival of breast patients with high stromal KDM2A expression (Table S3). These results suggested that expression of KDM2A in CAFs is a marker of poor prognosis in breast cancer.

**Table 1:** Correlation between stromal KDM2A expression and various clinicopathological factors.

Parameters	Category	No. of case	Stromal KDM2A expression		*P* value
			Low	High	
Age (years)	<60 years	138	69	69	1.000
	≧60 years	46	23	23	
Primary tumour (T)	T1	84	53	31	0.001^a^
	T2	85	36	49	
	T3–4	15	3	12	
Nodal status (N)	N0	114	64	50	0.034^a^
	N1–N2	70	28	42	
Stage	I	66	42	24	0.011^a^
	II	101	45	56	
	III	17	5	12	
Histological grade	Grade I	13	11	2	0.004^a^
	Grade II	129	55	74	
	Grade III	39	24	15	

^a^Statistically significant.

### Cancer-derived cytokines stimulate KDM2A expression in fibroblasts

**Fig. 2:**
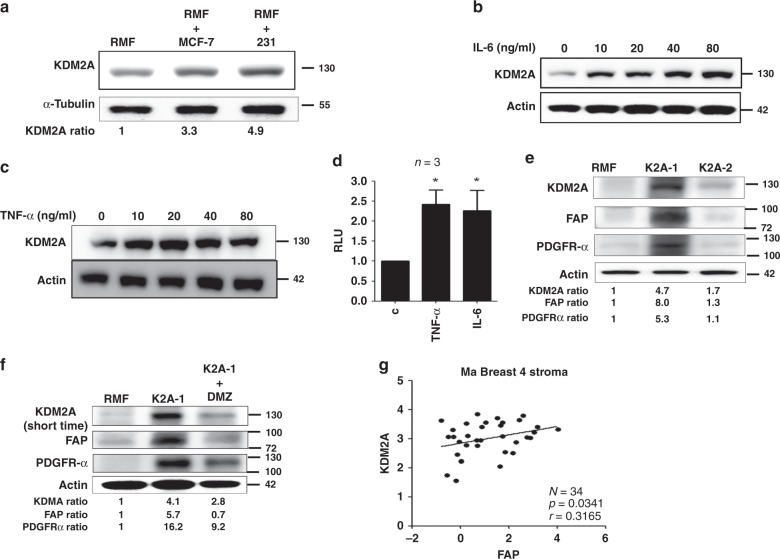
Cancer-derived cytokines upregulate KDM2A expression in normal fibroblasts and transform normal fibroblasts into CAFs. **a** Co-culture of normal human mammary fibroblasts (RMF-EG) with MCF-7 or MDA-MB-231 (231) breast cancer cells for 48 h and the expressions of KDM2A in RMF-EG cells were investigated by western blotting. **b** Normal RMF-EG mammary fibroblasts were treated with various doses of IL-6 and the KDM2A protein level was studied. **c** Normal RMF-EG mammary fibroblasts were treated with various doses of TNF-α and the KDM2A protein level was investigated. **d** RMF-EG cells were transfected with pEZX-PG04-KDM2A gene promoter construct and treated with recombinant IL-6 or TNF-α (20 ng/ml) for 24 h. The promoter activity was determined by using the Secrete-Pair Dual Luminescence Assay Kit. *P < 0.05. **e** Normal RMF-EG mammary fibroblasts were transfected with KDM2A expression vector and two independent stable clones K2A-1 and K2A-2 were established by antibiotics selection. Expression of KDM2A, FAP and PDGFR-α was investigated by western blotting. **f** The K2A-1 cells were treated without or with daminozide (2.5 μM) for 24 h and the protein level of KDM2A, FAP and PDGFR-α was compared. **g** The association between KDM2A and FAP expression in the tumour stroma of 34 breast tumours, obtained from the microarray data GSE145148, was analysed and a positive correlation was found (*P* = 0.0341).

We next addressed how KDM2A was upregulated in CAFs. Co-culture of MDA-MB-231 and MCF-7 breast cancer cells with immortalised normal human mammary fibroblasts (RMF-EG) increased KDM2A expression in the fibroblasts (Fig. 2a). It is possible that breast cancer cell-released factors may upregulate stromal KDM2A. Indeed, treatment of normal RMF-EG fibroblasts with IL-6 and TNF-α, two abundant cytokines in the conditioned medium of MDA-MB-231 cancer cells,^18^ increased KDM2A protein level in a dose-dependent manner suggesting these two cytokines may play a role in stimulating KDM2A expression in fibroblasts (Fig. 2b, c). Promoter activity assays demonstrated that IL-6 and TNF-α upregulated KDM2A via transcriptional activation (Fig. 2d). We ectopically expressed the KDM2A gene in normal RMF-EG fibroblasts and established two independent stable clones K2A-1 and K2A-2. K2A-1 cells exhibited a 4.7-fold increase in KDM2A protein level and these cells showed significant upregulation in fibroblast activation protein (FAP) and platelet-derived growth factor receptor-α (PDGFR-α), two biomarkers of CAFs. Conversely, K2A-2 cells showed a marginal increase in KDM2A, FAP and PDGFR-α suggesting these cells only exhibited partial CAF phenotype. These data suggested that KDM2A may transform normal mammary fibroblasts into CAFs in a dose-dependent fashion (Fig. 2e). Other CAF-associated genes including fibroblast growth factor 2 (FGF2) and C-C motif ligand 5 (CCL5) were expressed much higher in the K2A-1 cells than in the K2A-2 cells (Fig. S2). Increase of FAP and PDGFR-α in the fibroblasts was dependent on the enzymatic activity of KDM2A because a KDM2A inhibitor daminozide effectively reversed the increase (Fig. 2f). Interestingly, long-term incubation of diaminozide also decreased KDM2A protein level in the fibroblasts. It is possible that diaminozide affects enzymatic activity and protein stability in a time-dependent manner. Few gene expression profiles of breast cancer stroma had been reported. By analysing the microarray data (GSE145148) published in a previous study,^19^ we found that expression of FAP was positively associated with KDM2A (*p* = 0.0341, Fig. 2g). These results suggested that breast cancer-derived cytokines stimulate KDM2A expression in normal mammary fibroblasts and transform them into CAFs.

### KDM2A induces p53-dependent senescence in normal mammary fibroblasts

**Fig. 3:**
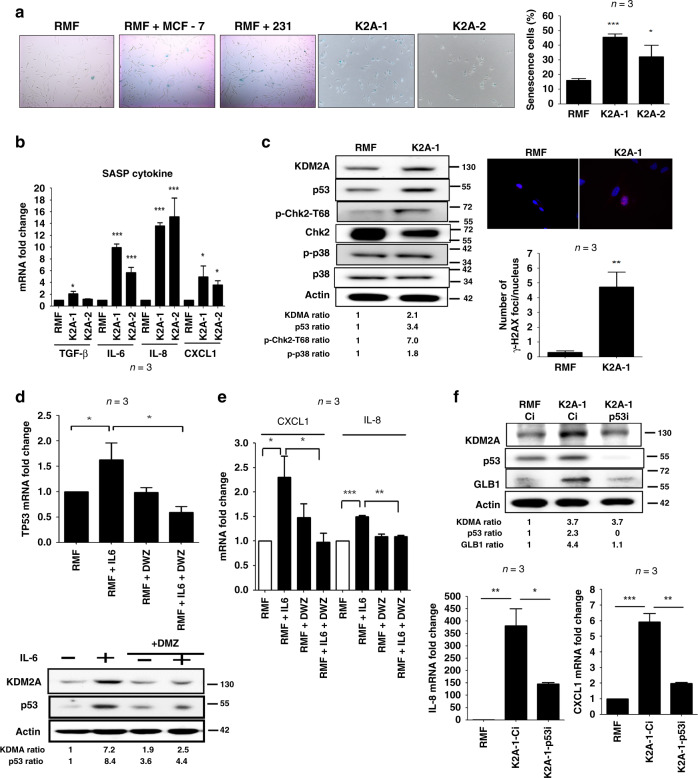
KDM2A induces p53-dependent senescence in normal human mammary fibroblasts. **a** Senescence of normal mammary fibroblasts RMF-EG cells co-cultured without or with breast cancer cells (MCF-7 or MDA-MB-231) and KDM2A-expressing K2A-1 and K2A-2 cells was investigated by β-galactosidase staining. The percentage of senescent cells of control RMF-EG, K2A-1 and K2A-2 was also quantified (*n* = 3). **b** The expression of senescence-associated secretary phenotype cytokines including TGF-β, IL-6, IL-8 and CXCL1 was detected by quantitative RT-PCR (*n* = 3). **c** The p53 protein level and the phosphorylation of Chk2 and p38 of KDM2A-overexpressing K2A-1 cells were compared to that of normal RMF-EG mammary fibroblasts. The phosphorylation of H2AX protein (γ-H2AX) was investigated by immunofluorescent staining and the number of γ-H2AX foci of RMF-EG and K2A-1 cells was compared (*n* = 3). **d** RMF-EG cells were pretreated without or with daminozide (2.5 μM) and stimulated with IL-6 (20 ng/ml) for 24 h. The mRNA and protein levels of p53 were investigated by quantitative RT-PCR and western blotting (*n* = 3). **e** RMF-EG cells were treated as described above and the expression of CXCL1 and IL-8 was quantified by quantitative RT-PCR (*n* = 3). **f** The K2A-1 cells were transfected with p53 siRNA (p53i) or control siRNA (Ci). After 24 h, cellular mRNAs and proteins were harvested. The protein level of KDM2A, p53 and β-galactosidase (GLB1) was investigated by western blotting and the mRNA level of IL-8 and CXCl1 was studied by quantitative RT-PCR (*n* = 3). *P < 0.05; **P < 0.01; ***P < 0.001.

When culturing the K2A-1 and K2A-2 stable clones, we found that the growth of these clones were slower than that of the control clones and the proliferation rate was reduced gradually. This led us to consider whether KDM2A induced senescence in the fibroblasts. Co-culture with MDA-MB-231 and MCF-7 breast cancer cells increased senescence in the normal RMF-EG fibroblasts as detected by the increase of β-galactosidase activity (Fig. 3a). Similarly, higher percentage of senescent cells were detected in the K2A-1 and K2A-2 clones (Fig. 3a). The expression of IL-6, IL-8, TGF-β and CXCL1, the senescence-associated secretary phenotype cytokines produced by senescent cells, was all increased in the K2A-1 and K2A-2 cells confirming the induction of senescence (Fig. 3b). We next tested whether the cytokines secreted by senescent cells could reciprocally stimulate cancer cell proliferation. Indeed, the conditioned medium of K2A-1 cells enhanced the proliferation of MDA-MB-231 cancer cells more efficiently than that of the normal RMF-EG cells (Fig. S3). When checking the cell cycle inhibitors p15, p16 and p53, we only found the increase of p53 protein in K2A-1 cells (Fig. 3c). The p38 signalling pathway, which has been reported to play a role in the induction of senescence, was marginally changed (Fig. 3c). Upregulation of p53 by KDM2A seemed to induce replication stress because the activity of CHK2 kinase and the phosphorylation of γ-H2AX proteins were significantly increased in the K2A-1 cells (Fig. 3c). The increase of p53 by KDM2A depended on its enzymatic activity because pre-treatment of daminozide totally abolished IL-6-induced upregulation of p53 mRNA and protein (Fig. 3d). Co-culture with MDA-MB-231 cells also increased p53 in the normal RMF-EG fibroblasts, which could be reversed by depletion of KDM2A (Fig. S4). In addition, the expression of senescence-associated IL-8 and CXCL1 was also significantly attenuated by treatment of daminozide (Fig. 3e). To further clarify the role of p53 in KDM2A-mediated senescence, we depleted p53 by siRNA in K2A-1 cells and found that p53 depletion did not affect KDM2A expression (Fig. 3f). However, it significantly decreased the expression of β-galactosidase (encoded by the GLB1 gene), IL-8 and CXCL1. These data suggested that the KDM2A-p53 axis triggered senescence in fibroblasts.

#### KDM2A upregulates PD-L1 expression in fibroblasts

**Fig. 4:**
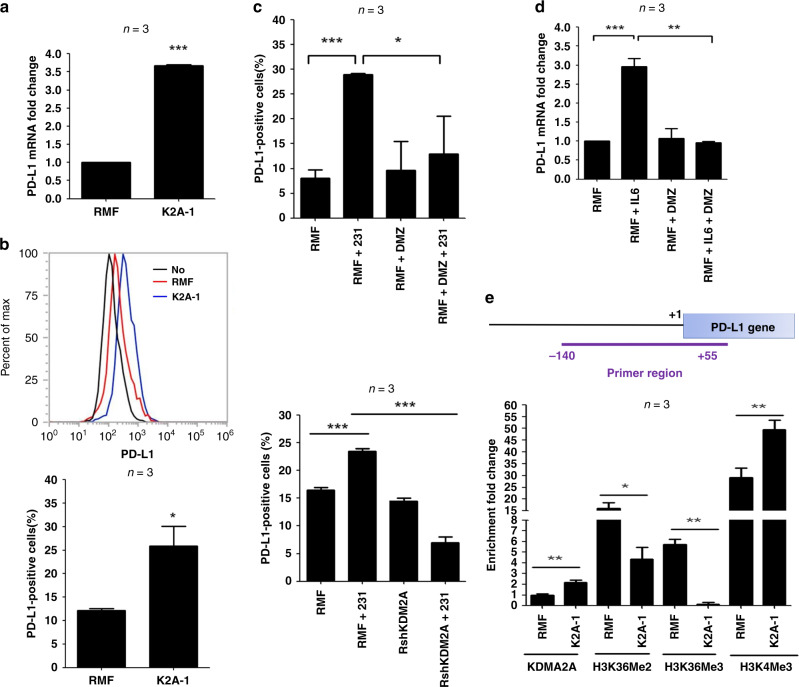
KDM2A upregulates PD-L1 expression in mammary fibroblasts via transcriptional activation. **a** Expression of PD-L1 mRNA in RMF-EG and K2A-1 cells was investigated by qRT-PCR. **b** Flow-cytometry analysis was done to study the cell surface PD-L1 in RMF-EG and K2A-1 cells. In addition, the percentage of PD-L1-positive cells was compared. **c** RMF-EG cells were co-cultured without or with MDA-MB-231 cells and incubated without or with daminozide for 24 h. The percentage of PD-L1-positive cells was compared in different experimental groups. In addition, RMF-EG and KDM2A-depleted RshKDM2A cells were co-cultured with MDA-MB-231 cells. After 24 h, PD-L1-positive cells were determined. **d** RMF-EG cells were pretreated without or with daminozide and then stimulated with IL-6 (20 ng/ml) for 24 h. Expression of PD-L1 was studied by qRT-PCR. **e** Genomic DNA was harvested from RMF-EG and K2A-1 cells and ChIP assays were performed by using various antibodies to investigate the binding of KDM2A to human PD-L1 gene promoter and the methylation status of H3K36 and H3K4 in the promoter. The genomic region amplified by the PCR primers was shown. *P < 0.05; **P < 0.01; ***P < 0.001.

Whether KDM2A plays any role in immune checkpoint regulation has not been reported. We compared the expression of various immune checkpoint molecules in the RMF-EG and K2A-1 cells and found that the mRNA and protein of programmed death-ligand 1 (PD-L1) was upregulated in the K2A-1 clone (Fig. 4a). In addition, the number of PD-L1-positive cells in the K2A-1 clone was increased compared to that of RMF-EG cells (Fig. 4b). Co-culture with MDA-MB-231 breast cancer cells enhanced PD-L1 expression in the RMF-EG fibroblasts, which could be significantly reduced by treatment of daminozide or knockdown of KDM2A (Fig. 4c). Inhibition of KDM2A activity by daminozide also inhibited IL-6-induced upregulation of PD-L1 mRNA in the RMF-EG cells (Fig. 4d). These data suggested that KDM2A may directly control PD-L1 expression. We performed ChIP assay to study the occupancy of KDM2A to the proximal promoter region of human PD-L1 gene and found the increase of KDM2A promoter binding in the K2A-1 cells (Fig. 4e). Methylation of H3K36 at the same region was decreased consisting with the finding that KDM2A is a H3K36 demethylase. In addition, trimethylation of H3K4, a transcriptional activation marker, was increased in the KDM2A promoter agreeing with the upregulation of PD-L1 expression. These data suggested an important role of KDM2A in immune checkpoint regulation in CAFs.

### Depletion of KDM2A in CAFs attenuates breast tumorigenesis in vivo

**Fig. 5:**
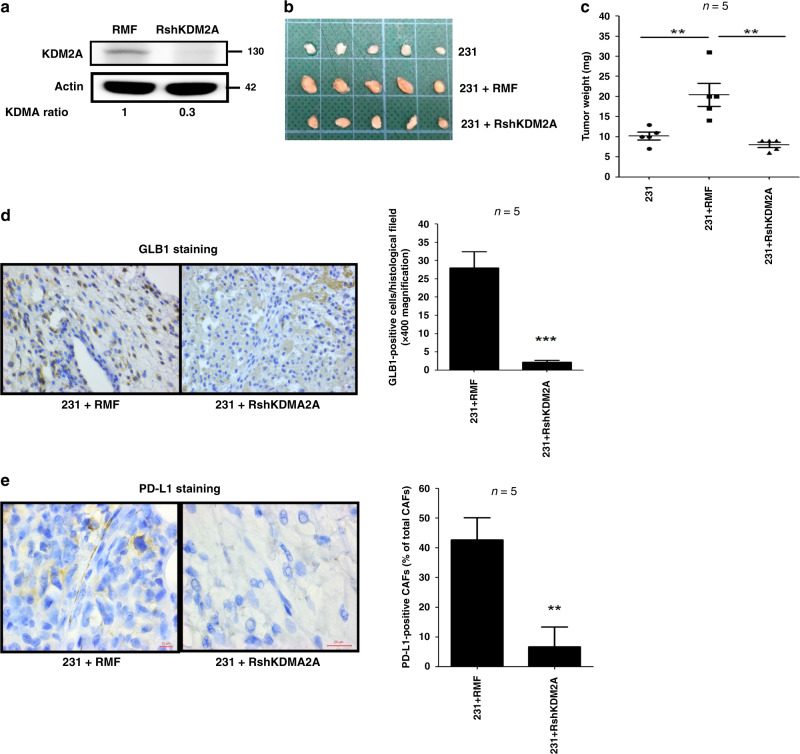
Stromal KDM2A promotes breast tumorigenesis and increases senescence. **a** RMF-EG cells were transfected with control or KDM2A shRNA. After 48 h, stable cell lines were selected by antibiotics. The KDM2A protein level in the control or KDM2A-depleted cell lines was compared by western blotting. **b** MDA-MB-231 breast cancer cells were co-injected without or with control or KDM2A-depleted RMF-EG mammary fibroblasts into the fourth mammary fat pads of 6-week-old female BALB/cAnN.Cg-Foxn1nu/CrlNarl mice. The tumours were harvested three weeks after injection. **c** Tumour weights were measured and were expressed as Mean ± SD. **d** The expression of β-galactosidase (GLB1) in tumour fibroblasts was investigated by immunohistochemical staining and the number of GLB1-positive fibroblasts in a 400× histological field was expressed as Mean ± SD. **e** The expression of PD-L1 in CAFs was studied by immunohistochemical staining. The number of PD-L1-positive CAFs was counted in a 400× histological field and was expressed as the percentage of the total CAFs counted. *P < 0.05; **P < 0.01; ***P < 0.001.

Whether KDM2A upregulation in CAFs promoted breast tumorigenesis was tested in animals. We repressed KDM2A expression in the normal RMF-EG fibroblasts by shRNA (Fig. 4a) and co-injected the control or KDM2A-depleted fibroblasts with MDA-MB-231 cancer cells into the fourth mammary fat pads of nude mice. As shown in Fig. 4b, c, co-injection with the normal RMF-EG fibroblasts promoted tumour growth while co-injection with the KDM2A-depleted RMF-EG cells did not. Our immunohistochemical staining showed that β-galactosidase was highly expressed in the stroma of the tumours established by co-injection of MDA-MB-231 and RMF-EG cells (Fig. 4d). The numbers of senescent fibroblasts were dramatically reduced in the tumours generated by co-injection of the KDM2A-depleted fibroblasts confirming our cell-based finding that cancer cell-released cytokines induced the senescence of CAFs via KDM2A. In addition, PD-L1-positive fibroblasts were only detected in the tumours established by co-injection of MDA-MB-231 and RMF-EG cells (Fig. 5d). These data suggested that KDM2A increased senescence and PD-L1 expression in fibroblasts in vivo as found in our cell-based assays.

## Discussion

In this study, we provide the first evidence that KDM2A is upregulated in human breast cancer stroma and is associated with poor clinical outcome. We also find that KDM2A triggers senescence and PD-L1 in CAFs to promote breast tumorigenesis. The tumour-promoting activity of senescent fibroblasts had been reported in previous studies. Yang et al. demonstrated that ovarian cancer cells with *Ras* mutation expressed abundant growth-regulated oncogene 1, which could act via paracrine mechanism to trigger fibroblast senescence to promote tumorigenesis.^20^ In a xenograft animal model, senescent fibroblasts had been shown to promote early tumour growth by secreting matrix metalloproteinases.^21^ In addition, senescent fibroblasts may fuel tumour growth by providing metabolic intermediates and coordinating amino acid availability.^22,23^ However, whether epigenetic modifying enzymes regulate cell senescence is largely unclear. We showed that cytokines released from breast cancer cells stimulate the transcription of KDM2A gene in fibroblasts, and KDM2A upregulation in fibroblasts increases p53 to promote cell senescence. A previous study demonstrated that ectopic expression of KDM2A or KDM2B bypasses replicative senescence by targeting the Rb pathway in mouse embryonic fibroblasts (MEFs).^8^ They also found that KDM2B increases p53 expression in MEFs. However, the activation of p53 pathway does not inhibit cell proliferation of MEFs, which is contradicted to our results that KDM2A-triggered p53 upregulation promotes senescence in human mammary fibroblasts. Interestingly, the study also reported that KDM2B could not prevent senescence in human primary fibroblasts. Therefore, KDM2A (or KDM2B) seems to play different roles in the regulation of proliferation and senescence of fibroblasts from different origin (human *vs* mouse) and differentiation status (embryonic *vs* mature fibroblasts). The induction of senescence in human mammary fibroblasts by KDM2A triggers senescence-associated secretary phenotype, which is accompanied with the release of a number of cytokines including IL-6, IL-8, CXCL1 etc. These cytokines reciprocally stimulate the proliferation of breast cancer cells as evidenced by the increase of cancer cell growth by the conditioned medium of KDM2A-overexpressing fibroblasts (Fig. S3).

Another novel finding of this study is the upregulation of PD-L1 by KDM2A. More than 70% of breast cancers contain immune cell infiltration in the stroma.^24^ However, the prognostic significance of immune cell infiltration in predicting the response to chemotherapy or immunotherapy is vary in different molecular subtypes. By using an elegant genetically engineered mouse model, Ruhland et al. reported that stromal senescence creates an immunosuppressive microenvironment to promote tumour formation.^25^ In the clinical setting, PD-L1 is detected in the epithelium or stroma in 30–60% of breast cancer and is a predictor of pathological complete response to neoadjuvant chemotherapy and immunotherapy.^26,27^ How PD-L1 is upregulated in the stroma of breast tumour is unclear. Here, we showed that KDM2A may directly bind to the promoter of PD-L1 gene to increase its expression in the mammary fibroblasts. The role of lysine demethylases in the regulation of immune checkpoint molecules has never been reported before. When this manuscript was under preparation, we found a study demonstrated that another histone demethylase lysine-specific demethylase 1 (LSD1) may suppress the expression of PD-L1 in breast cancer cells, and inhibition of LSD1 elicits anticancer immunity in breast cancer.^28^ In addition, combination of LSD1 inhibitor and PD-L1 antibody showed a more profound effect in the inhibition of tumour growth. Because LSD1 demethylates H3K4 and H3K9, two activation markers in the gene promoters, inhibition of LSD1 may lead to the increase of H3K4/H3K9 methylation and the restoration of PD-L1 expression. Conversely, KDM2A, which demethylates H3K36, may activate or repress the transcription of target genes. We demonstrated that KDM2A is a positive regulator of PD-L1. The distinct roles of LSD1 and KDM2A in the control of PD-L1 expression may be due to the difference of cells investigated. Qin et al. mainly studied the effect of LSD1 in breast cancer cells while we addressed the role of KDM2A in mammary fibroblasts.^28^ Whether lysine demethylases control immune checkpoint molecules in a cell type-dependent manner needs further characterisation.

Collectively, our study reveals a novel function of KDM2A in the regulation of senescence and PD-L1 expression in CAFs and elucidates the underlying mechanism by which stromal KDM2A promotes breast tumorigenesis.

## Supplementary information

Supplementary Figures 1-4 and supplementary Tables 1-3

## Data Availability

All data and materials generated and/or analysed during the current study are available from the corresponding author upon reasonable request.
