# Supplementary material for: Lysine demethylase 2A expression in cancer-associated fibroblasts promotes breast tumour growth
Source: Br J Cancer. 2020 Oct 7;124(2):484–93. doi: 10.1038/s41416-020-01112-z (PMC7852571; doi:10.1038/s41416-020-01112-z)
Supplement: Supplementary file 1 — Supplementary Figures 1-4 and supplementary Tables 1-3 [file 41416_2020_1112_MOESM1_ESM.pdf]

Figure S1

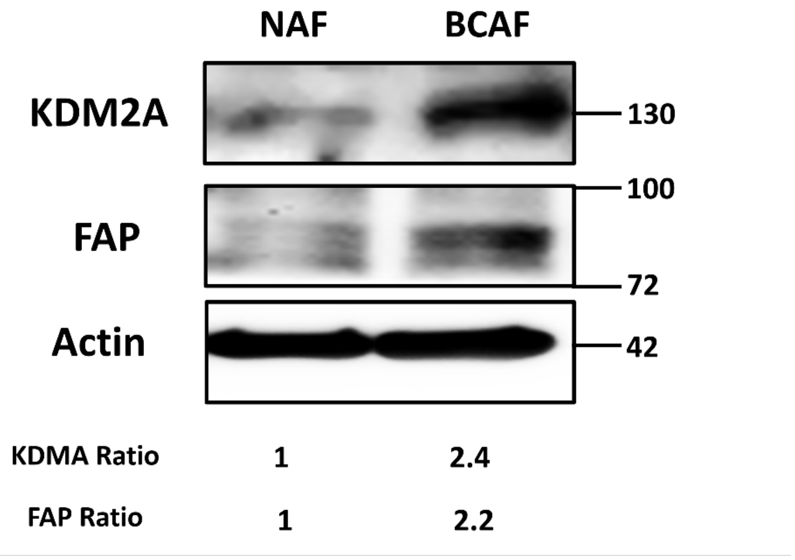

Fig. S1. Increase of KDM2A protein level in isolated cancer-associated fibroblasts of breast tumor. Fibroblasts isolated from non-tumor part (NAF) or tumor part (BCAF) in our previous study (Che JY et al., Breast Cancer Research 16:410, 2014) were recovered from frozen vials and the protein levels of KDM2A, FAP and Actin were investigated by Western blotting. The signal intensities of KDM2A and FAP were normalized to the signal intensities of actin.

Figure S2

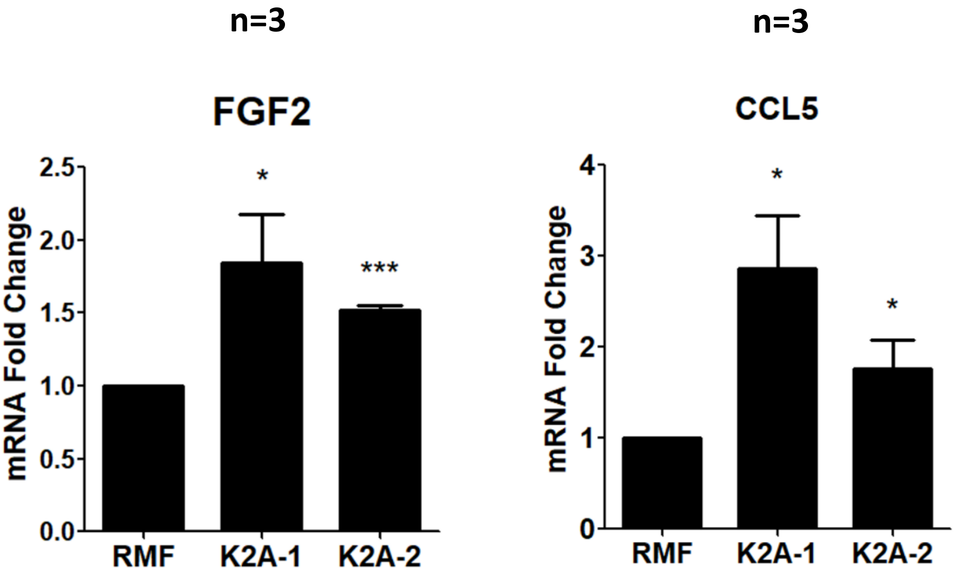

Fig. S2. Increased expression of CAF-associated genes (FGF2 and CCL5) in KDM2A-expressing stable clones.

Normal mammary fibroblasts (RMF-EG) were transfected with KDM2A expression vector and two stable cell clones K2A-1 and K2A-2 with different KDM2A expression were established by antibiotic selection. The expression of FGF2 and CCL5, two CAF-associated genes, was investigated by quantitative RT-PCR. (n=3, *p<0.05, ***p<0.001).

Figure S3

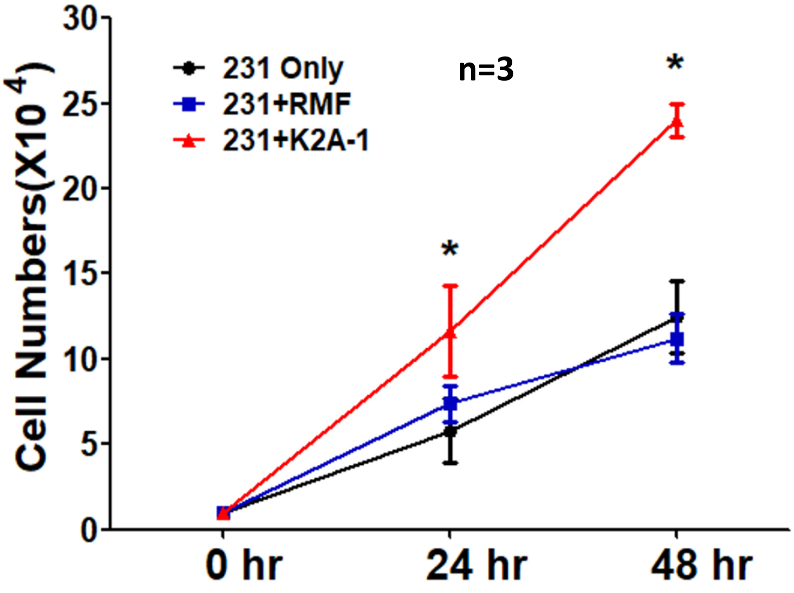

Fig S3. The conditioned medium of K2A-1 cells effectively stimulated the proliferation of MDA-MB-231 cancer cells.

The control medium or the conditioned media of RMF-EG and K2A-1 cells were collected. MDA-MB-231 cells were cultured with the media and the cell numbers of various treated groups were measured at 24 and 48 h after treatment. (n=3, $*p<0.05$).

Figure S4

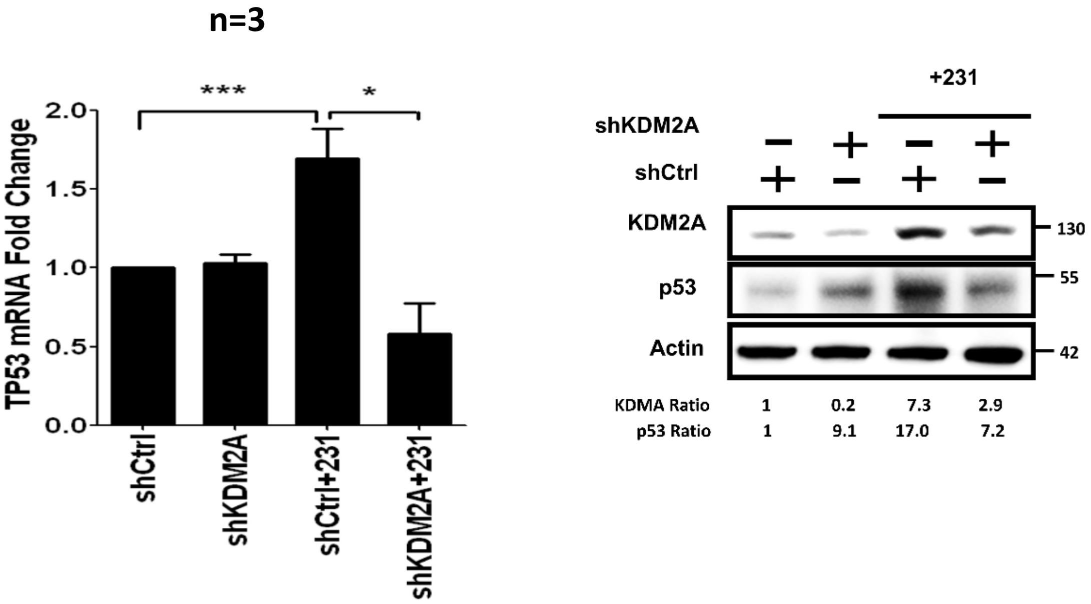

Fig. S4. Co-culture with MDA-MB-231 breast cancer cells upregulated p53 in RMF-EG fibroblasts in a KDM2A-dependent manner.

The RMF-EG fibroblasts were co-cultured with MDA-MB-231 cells in a trasnwell unit for 48 h as described in Methods. Total RNA and cellular proteins of fibroblasts were harvested and the mRNA and protein levels of p53 were investigated by quantitative RT-PCR and Western blotting.

Table S1. The Primer List

RT-PCR	
KDM2A-Forward	5'- CTCCCTTGAGCTTGGTTCTG-3'
KDM2A-Reverse	5'- AATCCACTTGGGTAGCAACG-3'
Actin-Forward	5'- TGTTACCAACTGGGACGACA-3'
Actin -Reverse	5'- GGGGTGTTGAAGGTCTCAAA-3'
TP53-Forward	5'- ACCTATGGAAACTACTTCCTGAAA -3'
TP53-Reverse	5'- CTGGCATTCTGGGAGCTTCA -3'
FAP-Forward	5'- TGGGAATATTACGCGTCTGTCTAC -3'
FAP-Reverse	5'- GATAAGCCGTGGTTCTGGTCA -3'
FGF2-Forward	5'- CCATCCAGTTCTGCTTTC-3'
FGF2 -Reverse	5'- GTTCGTTTCAGTGCCACATACC-3'
CCL5-Forward	5'- CGTGCCCACATCAAGGAG-3'
CCL5 -Reverse	5'- GGACAAGAGCAAGCAGAAAC-3'
TGF-β-Forward	5'- GGAAATTGAGGGCTTTCGCC -3'
TGF-β -Reverse	5'- CCGGTAGTGAACCCGTTGAT -3'
IL-6-Forward	5'- GACAGCCACTCACCTCTTCA -3'
IL-6 -Reverse	5'- TGCAGGAACTGGATCAGGAC -3'
IL-8-Forward	5'- AGACAGCAGAGCACACAAGC -3'
IL-8 -Reverse	5'- AATTTCTGTGTTGGCGCAGT -3'
CXCL1-Forward	5'- CTTGCCTCAATCCTGCATC -3'
CXCL1 -Reverse	5'- CCTTCTGGTCAGTTGGATTTG -3'

Table S2. Univariate log-rank analysis for disease-specific survival and metastasis-free survival

Parameters	Category	No. of case	DSS		MeFS	
			No. of event	*P*-value	No. of event	*P*-value
**Age (years)**	<60 years	138	13	0.8183	38	0.2071
	≧60 years	46	4		16	
**Primary tumor (T)**	T1	84	4	**0.0211***	9	**<0.0001***
	T2	85	11		34	
	T3-4	15	2		12	
**Nodal status (N)**	N0	114	7	**0.0019***	18	**<0.0001***
	N1-2	70	10		36	
**Stage**	I	66	2	**<0.0001***	4	**<0.0001***
	II	101	10		37	
	III	17	5		13	
**Histological grade**	Grade I	13	0	0.4385	1	0.0593
	Grade II	129	14		38	
	Grade III	39	3		15	
**Stromal KDM2A expression**	Low Exp.(<medium)	92	3	**0.0012***	14	**<0.0001***
	High Exp.(≧medium)	92	14		40	

*** Statistically significant**

Table S3. Multivariate survival analysis

Parameter	Category	DSS			MeFS		
		H.R	95% CI	*P*-value	H.R	95% CI	*P*-value
**Stage**	I	1	-	**<0.001***	1	-	**<0.001***
	II	3.604	0.785-16.554		5.593	1.963-15.935	
	III	16.831	3.187-88.894		18.077	5.755-56.784	
**Stromal KDM2A expression**	Low Exp.(<medium)	1	-	**0.018***	1	-	**0.001***
	High Exp.(≧medium)	4.564	1.291-16.132		2.749	1.474-5.127	
**Histological grade**	Grade I	-	-	-	1	-	**0.048***
	Grade II	-	-	-	2.388	0.319-17.865	
	Grade III	-	-	-	3.993	0.520-30.655	

***Statistically significant**
